# Language experience influences audiovisual speech integration in unimodal and bimodal bilingual infants

**DOI:** 10.1111/desc.12701

**Published:** 2018-07-16

**Authors:** Evelyne Mercure, Elena Kushnerenko, Laura Goldberg, Harriet Bowden‐Howl, Kimberley Coulson, Mark H Johnson, Mairéad MacSweeney

**Affiliations:** ^1^ UCL Institute of Cognitive Neuroscience London UK; ^2^ School of Psychology University of East London London UK; ^3^ School of Psychology University of Plymouth Plymouth UK; ^4^ Department of Psychology and Sports Sciences University of Hertfordshire Hatfield Hertfordshire UK; ^5^ Centre for Brain and Cognitive Development Birkbeck – University of London London UK; ^6^ Department of Psychology University of Cambridge Cambridge UK

## Abstract

Infants as young as 2 months can integrate audio and visual aspects of speech articulation. A shift of attention from the eyes towards the mouth of talking faces occurs around 6 months of age in monolingual infants. However, it is unknown whether this pattern of attention during audiovisual speech processing is influenced by speech and language experience in infancy. The present study investigated this question by analysing audiovisual speech processing in three groups of 4‐ to 8‐month‐old infants who differed in their language experience: monolinguals, unimodal bilinguals (infants exposed to two or more spoken languages) and bimodal bilinguals (hearing infants with Deaf mothers). Eye‐tracking was used to study patterns of face scanning while infants were viewing faces articulating syllables with congruent, incongruent and silent auditory tracks. Monolinguals and unimodal bilinguals increased their attention to the mouth of talking faces between 4 and 8 months, while bimodal bilinguals did not show any age difference in their scanning patterns. Moreover, older (6.6 to 8 months), but not younger, monolinguals (4 to 6.5 months) showed increased visual attention to the mouth of faces articulating audiovisually incongruent rather than congruent faces, indicating surprise or novelty. In contrast, no audiovisual congruency effect was found in unimodal or bimodal bilinguals. Results suggest that speech and language experience influences audiovisual integration in infancy. Specifically, reduced or more variable experience of audiovisual speech from the primary caregiver may lead to less sensitivity to the integration of audio and visual cues of speech articulation.

**RESEARCH HIGHLIGHTS**

- Increased attention to the mouth was observed between 4 and 8 months in monolinguals and unimodal bilinguals, but not in bimodal bilingual infants with Deaf mothers.
- Audiovisual incongruence of speech articulation increased visual attention to the mouth in older (6.6–8 months), but not younger (4–6.5 months) monolinguals.
- Audiovisual incongruence did not influence face scanning in bilingual infants (4–8 months), which may indicate increased tolerance to articulatory inconsistencies and imprecision.
- Multi‐modal speech processing in infancy requires sufficient audiovisual speech experience.

## 1 INTRODUCTION

A few weeks after birth and several months before they begin producing canonical babbling, infants can perceptually integrate audio and visual cues of speech articulation. Indeed, from 2 months of age, infants look longer at a face articulating a phoneme that they can hear as opposed to a face articulating a different phoneme (Kuhl & Meltzoff, 1982; Patterson & Werker, 1999, 2002, 2003). Further evidence of early audiovisual integration abilities in infancy can be found from the McGurk effect (Burnham & Dodd, 2004; Kushnerenko, Teinonen, Volein, & Csibra, 2008; McGurk & MacDonald, 1976; Rosenblum, Schmuckler, & Johnson, 1997). In this well‐documented phenomenon, adults automatically integrate incongruent audio and visual cues of articulation into an illusory percept. For some phoneme combinations (for example, a visual /ga/ and an auditory /ba/), adults do not typically perceive the incongruence of the audiovisual cues, but ‘fuse’ them into the closest English phoneme (usually in this case /da/ or /δa/). In other (non‐fusible) combinations of incongruent cues (for example a visual /ba/ and auditory /ga/), the resulting phoneme is perceived to be strange or illegal in English (usually in this case /bga/). Measuring ERPs in 5‐month‐old infants, Kushnerenko et al. (2008) observed an ERP mismatch response when presenting incongruent audiovisual cues of articulation that would typically be non‐fusible in adults (e.g., visual /ba/ + audio /ga/ = /bga/), but not for incongruent audiovisual combinations that are typically fusible into a legal English phoneme in adults (e.g., visual /ga/ + audio /ba/ = /da/). This ERP finding suggests that young infants can perceive audiovisual incongruence similarly to adults, although this integration may be less robust in infants than in adults and more restricted to certain stimulus combinations (Desjardins & Werker, 2004). Moreover, it has been observed that 6‐ and 9‐month‐old infants could detect audiovisual incongruences in a non‐native phonological contrast, but this sensitivity was not observed in 11‐month‐old infants (Danielson, Bruderer, Kandhadai, Vatikiotis‐Bateson, & Werker, 2017). These results suggest that audiovisual speech perception follows a pattern of perceptual attunement in the first year of life. This phenomenon, initially described for auditory speech perception, is characterized by a decline in sensitivity to non‐native consonant contrasts and improvement in sensitivity to native contrasts (Kuhl, Tsao, Liu, Zhang, & Boer, 2001; Narayan, Werker, & Beddor, 2010; Werker & Gervain, 2013).

Another interesting phenomenon in infancy is a progressive shift with age in infants’ focus of attention when looking at a talking face. Lewkowicz and Hansen‐Tift (2012) noted that young infants usually focus most of their attention on the eyes of a face talking in the infant's native language or a non‐native language. Between 4 and 8 months babies gradually shift their focus of attention to the mouth of a talking face and preferential looking to the mouth is observed at 8 and 10 months for native and non‐native languages. Interestingly, 12‐month‐old infants show preferential looking to the mouth for faces talking in a non‐native language, while no preference for the eyes or mouth is observed for the native language. Converging results have been obtained by Tomalski and colleagues (2013). This attentional shift towards the mouth around 4–8 months reflects a new stage in infants’ language development, occurring at around the same time as the onset of canonical babbling and accompanied by developmental changes in brain responses (Kushnerenko, Tomalski, Ballieux, Ribeiro et al., 2013). Increased looking time to the mouth at this age may reflect increased attention to the multisensory nature of audiovisual speech. Moreover, when presented with inconsistent audiovisual stimuli, infants who develop better language skills at 14–16 months looked less at the mouth (and more at the eyes) at 6–9 months than infants who develop poorer language skills at 14–16 months (Kushnerenko, Tomalski, Ballieux, Potton et al., 2013). These findings suggest that infants showing more advanced language development were better at ignoring inconsistent cues of visual articulation and focusing on the additional social cues provided by the eyes when presented with incongruent audiovisual articulation.

Interestingly, bilingual infants who experience two different phonological systems show different visual scanning patterns of talking faces (Pons, Bosch, & Lewkowicz, 2015). At 4 months, they show increased attention to the mouth compared to monolinguals, which results in no preference for the mouth or eyes of a talking face in this group. A strong preference for the mouth is observed in this group at 8 months and at 12 months for native and non‐native languages. Increased attention to the mouth was also observed for faces displaying non‐linguistic emotional movements in 8‐month‐old bilinguals compared to monolinguals (Ayneto & Sebastian‐Galles, 2017). In other words, bilingual infants’ attention to the mouth is increased compared to monolinguals. That is, they may be able to take advantage of audiovisual associations of the articulatory signal at an earlier age and for a longer period than monolingual infants. Furthermore, 8‐month‐old bilinguals are better at distinguishing two different languages when silently articulated than monolingual infants of the same age (Sebastián‐Gallés, Albareda‐Castellot, Weikum, & Werker, 2012; Weikum et al., 2007). These findings suggest that language experience influences the representation of audiovisual speech and that infants learning two auditory phonological systems may be more sensitive to visual cues of articulation. However, it remains unclear how the representation of audiovisual speech would be affected by a reduced experience of audiovisual speech, as experienced by hearing infants of Deaf^1^ mothers.

If a Deaf mother uses a signed language such as British Sign Language (BSL) as her preferred mode of communication, the speech and language experience of her hearing infant is likely to differ from that of hearing infants of hearing mothers in several ways. (1) First, hearing infants with Deaf mothers experience a signed language processed mainly in the visual modality (e.g. BSL), and a spoken language processed mainly in the auditory modality (e.g. spoken English). Because these infants are exposed to two languages in different modalities—signed and spoken—they are often referred to as ‘bimodal bilinguals’. On the other hand, the term ‘unimodal bilinguals’ is commonly used in the literature to describe bilinguals exposed to two spoken languages. This terminology does not deny the multimodal nature of speech perception and the crucial importance of audiovisual speech integration, but aims to emphasize their difference from bimodal bilinguals who are exposed to two languages processed in very different sensory systems. (2) Moreover, the language produced by a Deaf mother to and around her infant is likely to comprise less audiovisual spoken language than that of a hearing mother. A Deaf mother may use sign language in many of her daily interactions in the presence of her infant. Many Deaf signing individuals also use speech to communicate with hearing people, but the extent to which they actually ‘voice’ their speech and produce sound, as opposed to silently mouth, is extremely variable (Bishop & Hicks, 2005). A Deaf mother may use both signed and spoken language when addressing her infant, but spoken utterances by Deaf mothers are reduced in length and frequency compared to those of hearing mothers (Woll & Kyle, 1989). (3) Also, the lack of auditory feedback can affect speech production in Deaf adults. The execution of phonological distinctions has been observed to be less precise when produced by Deaf than hearing adults and changes in fine pitch control have also been noted (Lane & Webster, 1991; Waldstein, 1990). These speech characteristics could make the model offered by Deaf mothers less accessible and more difficult to learn for their hearing infants. (4) Finally, infants with Deaf mothers often experience visual forms of communication that require visual attention. Although visual speech is important to language development, it is not required for auditory speech comprehension. In contrast, sign language communication requires visual attention to the signer. Infants with and without experience of sign language, as well as adult signers, focus mainly on the face and not the hands when perceiving sign language (De Filippo & Lansing, 2006; Emmorey, Thompson, & Colvin, 2008; Muir & Richardson, 2005; Palmer, Fais, Golinkoff, & Werker, 2012). This increased attention to the face is the hypothesized mechanism for enhancement of certain aspects of face processing in Deaf and hearing signers compared to non‐signers (Bettger, Emmorey, McCullough, & Bellugi, 1997; Emmorey, 2001; McCullough & Emmorey, 1997; Stoll et al., 2018). Visual attention is critical to sign language communication. As a result Deaf mothers have been observed to use various strategies to obtain visual attention from their child, such as moving or signing in their child's existing focus of attention (Woll & Kyle, 1989). These strategies may lead to increased visual attention to faces in bimodal bilingual infants.

To our knowledge, audiovisual speech integration has never been studied in bimodal bilingual infants. These infants represent a unique opportunity for studying the role of speech and language experience in the development of speech processing in infancy. The present study investigated visual processing of faces articulating syllables with congruent, incongruent and silent auditory tracks. Three groups of 4‐ to 8‐month‐old infants with different language experience were compared: monolinguals, unimodal bilinguals and bimodal bilinguals. It was hypothesized that unimodal bilinguals would be more mature than monolinguals in audiovisual speech integration. Their increased attention to visual cues of articulation could allow unimodal bilinguals to take better advantage of the audiovisual associations of the articulatory signal. Unimodal bilinguals were expected to be more sensitive to audiovisual incongruences than monolinguals and to show increased attention to the mouth regardless of age. It was hypothesized that bimodal bilinguals would demonstrate increased attention to faces, but immaturities in their audiovisual speech integration because of their reduced audiovisual speech experience. More specifically, bimodal bilinguals were expected to show less sensitivity to audiovisual incongruences in syllable articulation and experience a delay in the age of their shift of visual attention to the mouth.

## 2 METHOD

### 2.1 Participants

Seventy‐three hearing infants between 4 and 8 months contributed data to the present study. A further 21 infants participated in the study but were excluded due to equipment malfunction or failure to calibrate (*n* = 4), withdrawal (*n* = 1), failure to reach looking time criteria (*n* = 14; see Data analyses) or prior inclusion of a sibling (*n* = 2). Infants were from three groups with different language experience: 28 monolingual infants with hearing parents (12 girls, mean age = 6.2 months), 22 unimodal bilingual infants with hearing parents (8 girls, mean age = 6.1 months) and 23 bimodal bilingual infants with a Deaf mother (14 girls; mean age = 6.3 months). Age did not differ between groups (*F*(2) = 0.182; *p* = 0.834). Monolingual infants were only exposed to English. Both parents were hearing and only used one language. Unimodal bilinguals were frequently and regularly exposed to English and one or more additional spoken language(s). The combination of languages varied between infants and there were 18 different additional languages in this study. All infants in this group had a hearing bilingual/multilingual mother. Fifteen unimodal bilingual infants also had a bilingual/ multilingual father, and seven had a monolingual father. None reported hearing deficits in any immediate family members. Exposure to each language was estimated by using an English adaptation of the language exposure questionnaire designed by Bosch and Sebastián‐Gallés (1997) (Byers‐Heinlein, 2009). Unimodal bilinguals were exposed to English on average 48% of the time (standard deviation = 24). Infants with hearing parents were contacted from the Birkbeck Babylab database of volunteers recruited from advertisements at mum‐and‐baby groups, parenting websites and publications. Bimodal bilinguals were frequently and regularly exposed to BSL and English.^2^ All infants in this group had a Deaf mother using BSL as her preferred mode of communication. Nineteen bimodal bilinguals also had a second severely/profoundly D/deaf parent, three had a second parent who was hearing or had mild hearing loss, and one had a single Deaf mother. Bimodal bilinguals were exposed to English on average 42% of the time (standard deviation = 22). There was no difference in language exposure between the two groups of bilinguals (*p* = 0.376). Bimodal bilinguals were recruited through social media and websites specifically aimed at the Deaf community. Most infants were born at term (37 to 42 weeks gestation), except for two infants born slightly before term (34–36weeks) (one monolingual and one unimodal bilingual: for these infants a corrected age was used). Infants had no hearing problems (except for one infant's mother reporting glue ear) or vision problems (except for one infant's mother reporting a suspected squint), no history of seizure or other serious mental or physical conditions according to their parents. Deaf families were geographically spread across the whole of Great Britain, while infants with hearing parents came mostly from London and surrounding areas. Travel expenses were reimbursed, and a baby T‐shirt and certificate of participation were offered to families. All parents gave informed consent prior to participation, after explanations of the study in English or BSL depending on the parents’ preferred mode of communication by fluent members of the research team. This study was approved by UCL and Birkbeck Research Ethics Committees and conforms to the Declaration of Helsinki.

### 2.2 Procedure

Infants were invited to participate in the larger *Speak & Sign* research protocol, including a functional near infrared spectroscopy task (investigating brain activation in response to infant‐directed spoken and sign language), three eye‐tracking tasks (the McGurk task reported here, as well as tasks investigating face orientation and eye gaze perception) and behavioural measures (the Mullen Scales of Early Learning and videos of parent–child interaction). The whole protocol usually required between 1.5 to 3 hours per infant, including resting, napping, and feeding time. Only data from the McGurk task are reported in the present article.

During the McGurk task, infants sat on their parent's lap in a dimly lit room about 60 centimetres from a TobiiT120 eye‐tracker (17‐inch diameter, screen refresh rate 60 Hz, ET sampling rate of 60 Hz, spatial accuracy < 1̊). Infant gaze position was calibrated with colorful animations using a 5‐point routine. Infant's gaze and behaviour was monitored throughout the study via camera and Tobii Studio LiveViewer. The parent's view of the stimulus monitor was obscured to avoid interference with infant behaviour.

### 2.3 Stimuli

Infants were presented with short videos of a female native English speaker repeatedly articulating /ba/ or /ga/. These stimuli have been used previously and are described in detail elsewhere (Kushnerenko et al., 2008; Kushnerenko, Tomalski, Ballieux, Ribeiro et al., 2013; Tomalski, Moore et al., 2013). There were five different experimental conditions: (1) congruent audiovisual /ba/; (2) congruent audiovisual /ga/; (3) fusible incongruent: audio /ba/ and visual /ga/, leading to the illusion of a perceptual fusion in adults (experienced as /da/ or /δa/); (4) non‐fusible incongruent: audio /ga/ and visual /ba/, leading to the non‐English percept ‘bga’ in adults, and (5) silent articulation (visual /ba/ or /ga/ presented without any auditory cues). Incongruent audiovisual conditions were created by dubbing audio files of one video onto a different one. Sound onset was adjusted in each articulated syllable to 360 ms from stimulus onset, with the auditory syllable lasting 300 ms. Each visual syllable lasted 760 ms with 10 repetitions presented in each trial (7,600 ms/trial). The face in the videos subtended approximately 14̊ of horizontal visual angle × 22̊ of vertical visual angle. Infants viewed 10 trials (two trials per condition) in a single fixed order block, in which the first two and last two trials were the congruent /ba/ and congruent /ga/. No effects of trial order were previously observed on looking behaviour in the same paradigm (Tomalski, Ribeiro et al., 2013). Before each trial, the infant's attention was attracted to the centre of the screen by presentation of a colourful animation with sound, which was terminated by the experimenter as soon as the infant focused on it.

### 2.4 Data analysis

**Figure 1:**
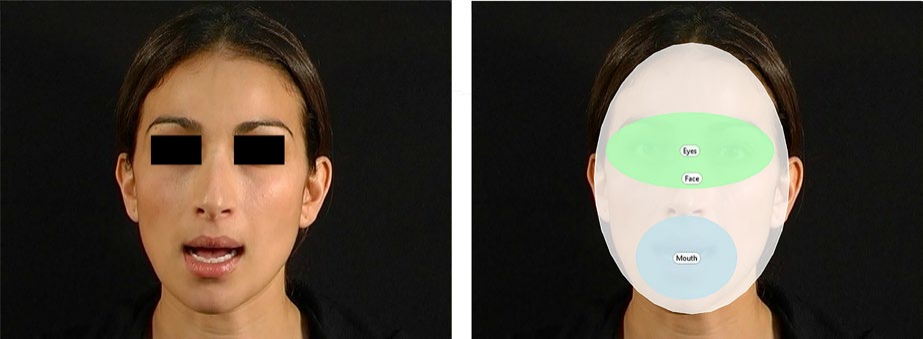
Example of stimuli and regions of interest

For each trial, the total looking time was extracted using Tobii Studio in three regions of interest: eyes region (oval shape of maximum dimension 285 × 128 pixels), mouth region (oval shape of maximum dimension 171 × 142 pixels) and entire face (oval shape of maximum dimension 332 × 459 pixels) (see Figure 1). Data were excluded in trials where infants looked at the entire face for less than 3 seconds. Only infants with at least 7 good trials out of 10 were included for analyses. Infants had on average 9.29 (standard deviation = 1.04) included trials, and there were no differences between groups in the number of included trials (*F*(2) = 1.690; *p* = 0.192). A Mouth‐to‐Face and Eyes‐to‐Face ratio was calculated for each condition and each participant.

## 3 RESULTS

### 3.1 Looking time to the entire face

A univariate ANOVA was performed on looking times to the entire face with 3 Groups and Included Trials as a covariate. A significant effect of Included Trials [*F*(1) = 17.385; *p* < 0.001; ƞ^2^ = 0.201] was found, suggesting, as would be expected on the basis of our exclusion criterion, that babies with more included trials generally spent longer looking at faces (*r* = 0.490; *p* < 0.001). A significant Group effect [*F*(2) = 5.023; *p* = 0.009; ƞ^2^ = 0.127] was also observed. Bimodal bilinguals (*p* = 0.001) and to some extent unimodal bilinguals (*p* = 0.076) spent more time looking at faces than monolinguals. There was no difference between the two groups of bilinguals (*p* = 0.573). Looking time to the entire face did not correlate with percentage of English exposure in the two groups of bilinguals (*r* = −0.005; *p* = 0.975).

### 3.2 Correlations of Mouth‐to‐Face ratio and Eyes‐to‐Face ratio with Age

**Figure 2:**
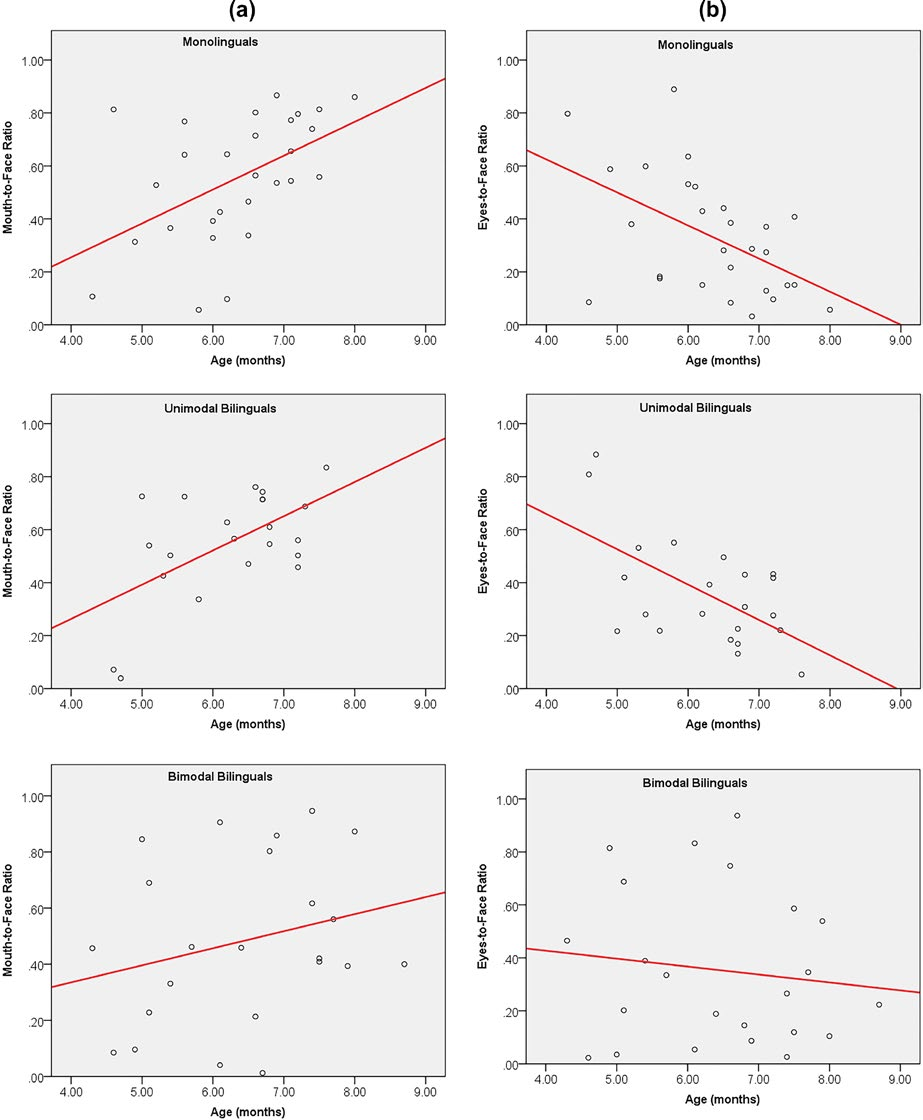
Individual (A) Mouth‐to‐Face ratio and (B) Eyes‐to‐Face ratio as a function of their age in each group

Correlations were assessed in each group between Mouth‐to‐Face ratio and the infant's age as well as between Eyes‐to‐Face ratio and the infant's age to assess the presence of an attentional shift from eyes to mouth in each group. For monolinguals, a highly significant positive correlation was found between Age and Mouth‐to‐Face ratio (*r* = 0.501; *p* = 0.006), as well as a highly significant negative correlation between Age and Eyes‐to‐Face ratio (*r* = −0.507; *p* = 0.005) (see Figure 2). Similar results were obtained for unimodal bilinguals, with a significant positive correlation between Age and Mouth‐to‐Face ratio (*r* = 0.524; *p* = 0.010), as well as a significant negative correlation between Age and Eyes‐to‐Face ratio (*r* = −0.522; *p* = 0.011). In contrast, for bimodal bilinguals no correlation between Age and Mouth‐to‐Face ratio (*r* = 0.241; *p* = 0.256) or Eyes‐to‐Face ratio (*r* = −0.128; *p* = 0.550) was observed. The difference between these correlation scores in monolinguals and bimodal bilinguals was non‐significant for Mouth‐to‐Face ratio (Fisher's *Z*‐test: *p* = 0.154), but nearly significant for Eyes‐to‐Face ratio (Fisher's *Z*‐test: *p* = 0.076). A similar pattern was observed in the comparison between unimodal and bimodal bilinguals (Fisher's *Z*‐test for mouth region: *p* = 0.147; for the eyes region: *p* = 0.079).

### 3.3 Effects of audiovisual congruency on Mouth‐to‐Face and Eyes‐to‐Face ratio

**Figure 3:**
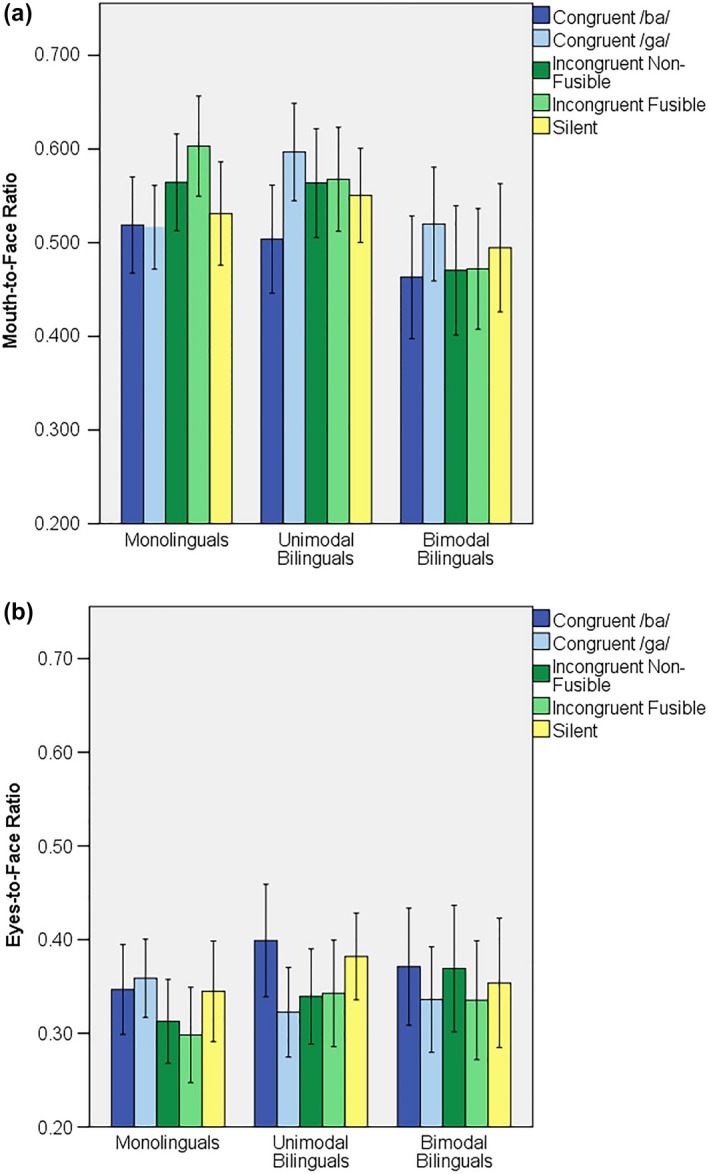
Percentage of time looking at the (A) mouth and (B) eyes in each condition and group. Error bars indicate standard error

A repeated measures ANOVA was performed on Mouth‐to‐Face and Eyes‐to‐Face ratios with 5 Conditions (Congruent /ba/, Congruent /ga/, Incongruent Non‐Fusible, Incongruent Fusible and Silent) × 2 Regions (Mouth and Eyes) × 3 Groups (Monolinguals, Unimodal Bilinguals and Bimodal Bilinguals), while Age and Included Trials were added as covariates (see Figure 3). A significant Region × Age interaction [*F*(1, 66) = 12.068; *p* = 0.001; ƞ^2^ = 0.155] was observed, which confirmed a general attentional shift from the eyes to the mouth with age. No significant Condition effect or interaction were observed.

To explore the impact of audiovisual congruency on looking times to the mouth, data were merged into Congruent (/ba/ + /ga/) and Incongruent articulations (Fusible + Non‐Fusible).^3^ Mouth‐to‐Face ratios were analysed in an ANOVA with 2 Congruency Conditions × 3 Groups, while Age and Included Trials were added as covariates. Age was the only significant factor [*F*(1, 68) = 13.01; *p* = 0.001; ƞ^2^ = 0.161].

**Figure 4:**
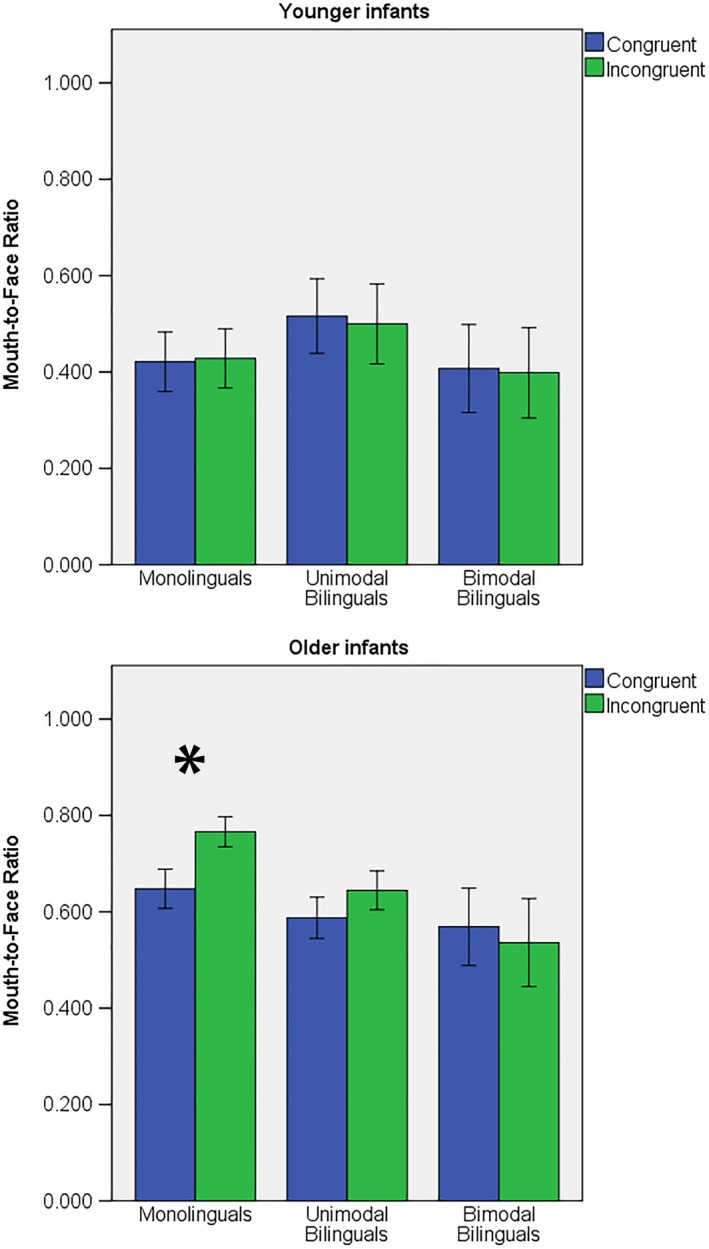
Mouth‐to‐Face ratio in congruent and incongruent audiovisual articulations in younger and older infants of each group. Error bars represent standard error

Given (1) the robust age effect in these analyses, (2) the fact that very different patterns of visual scanning of talking faces have been observed in infants before and after 6 months (Lewkowicz & Hanson‐Tift, 2012; Tomalski, Ribeiro et al., 2013), and (3) the fact that differences between monolinguals and unimodal bilinguals differ greatly across different age groups in infancy (Pons et al., 2015), two age groups were created using a median split. Younger infants were 4 to 6.5 months (15 monolinguals, 11 bimodal bilinguals and 12 unimodal bilinguals), while older infants were 6.6 to 8 months (13 monolinguals, 12 bimodal bilinguals, 10 unimodal bilinguals). An ANOVA was performed in each age group with 2 Congruency Conditions × 3 Groups and Included Trials as a covariate (see Figure 4). In the younger group of infants, there was no Congruency effect or interaction with Group. In the older group, Congruency × Group [*F*(2, 31) = 3.495; *p* = 0.043; ƞ^2^ = 0.184] was significant. In older monolinguals, incongruent articulations were associated with a higher percentage of looking time at the mouth [*F*(1, 12) = 28.844; *p* < 0.001; ƞ^2^ = 0.706]. Unimodal bilinguals [*F*(1, 9) = 1.828; *p* = 0.209; ƞ^2^ = 0.169] and bimodal bilinguals [*F*(1,11) = 0.360; *p* = 0.561; ƞ^2^ = 0.032] showed no Congruency effect.

These same analyses performed with 5 Conditions (Congruent /ba/, Congruent /ga/, Incongruent Non‐Fusible, Incongruent Fusible and Silent) × 3 Groups (Monolinguals, Unimodal Bilinguals and Bimodal Bilinguals) revealed no significant effect or interaction in the younger group. In the older group, a significant Condition × Group interaction was found [*F*(8, 60) = 2.332; *p* = 0.030; ƞ2 = 0.116]. A one‐way ANOVA examining Condition in each of the older groups separately showed a significant Condition effect in monolinguals [*F*(4, 9) = 5.839; *p* = 0.013; ƞ^2^ = 0.722], but not in unimodal bilinguals [*F*(4, 7) = 1.419; *p* = 0.321; ƞ^2^ = 0.448], nor in bimodal bilinguals [*F*(4, 7) = 1.121; *p* = 0.418; ƞ^2^ = 0.391]. Post‐hoc *t* tests revealed that older monolinguals looked longer at the mouth when processing incongruent than congruent articulations (all 4 *p* < 0.05). No difference was found between congruent /ba/ and congruent /ga/ (*p* = 0.777), between the two incongruent conditions (*p* = 0.159) or between silent and non‐silent conditions (all *p* > 0.05).

## 4 DISCUSSION

The present study aimed to assess the role of language experience in shaping the development of audiovisual speech integration. Visual attention to faces articulating syllables with congruent, incongruent and silent auditory tracks was studied in three groups of 4‐ to 8‐month‐old infants with different language experience: monolingual infants, unimodal bilingual infants (infants exposed to two spoken languages) and bimodal bilingual infants (hearing infants with Deaf parents).

Monolingual infants showed a decrease in looking time to the eyes and an increase in looking time to the mouth of talking faces between 4 and 8 months. This result is congruent with the observation of Lewkowicz and Hansen‐Tift (2012) and suggests that this attentional shift to the mouth around 6 months is not specific to faces articulating sentences in infant‐directed language, but can also be observed for faces articulating syllables. Monolinguals also increased their sensitivity to audiovisual incongruences between 4 and 8 months. While the youngest group (4 to 6.5 months) did not show any differences in scanning patterns between audiovisually congruent and incongruent syllables, older infants (6.6 to 8 months) looked longer at the mouth when the audiovisual cues of articulation were incongruent than congruent, indicating surprise or novelty. Older monolinguals also tended to look longer at the mouth in the case of non‐fusible incongruent than fusible incongruent articulation, although this difference did not reach significance. This finding is generally consistent with previous results (Kushnerenko et al., 2008; Kushnerenko, Tomalski, Ballieux, Potton et al., 2013; Tomalski, Ribeiro et al., 2013). While the difference in brain responses was specific to the *non‐fusible* condition in younger infants (5‐month‐olds) (Kushnerenko et al., 2008), the looking behaviour of older infants (8–9 months) revealed increased attention to the speaker's mouth for both the fusible and the non‐fusible incongruent conditions compared to the congruent condition (Tomalski, Ribeiro et al., 2013). Although infants can distinguish audiovisual congruencies from 2 months of age (Kuhl & Meltzoff, 1982; Patterson & Werker, 1999, 2002, 2003), the present results suggest that sensitivity to audiovisual cues of articulation for native phonemes still develops up until the second half of the first year in monolinguals.

Unimodal bilingual infants have different experience of speech and language compared to monolinguals as they are exposed to two different auditory phonological systems. Like monolinguals, unimodal bilingual infants showed an attentional shift towards the mouth and away from the eyes between 4 and 8 months. The predicted increase in attention to the mouth compared to monolinguals was not observed in this study, but a general increase in looking time to the entire face was observed. Increased attention to the mouth might have been observed if a larger proportion of younger infants (around 4 months) had been part of the present sample. Indeed, Pons and colleagues (2015) found increased attention to the mouth in unimodal bilinguals compared to monolinguals at 4 months, while no difference was observed at 8 months. Similarly, the data from our youngest group of infants (4 to 6.5 months, *n* = 11) show a trend towards an increase in looking time to the mouth in unimodal bilinguals compared to monolinguals. It is also important to note that the stimuli used in the present study were very different from the ones used by Pons and colleagues. Fluent infant‐directed speech may elicit different levels of attention and different processing strategies than repeated syllables.

It was also hypothesized that unimodal bilinguals would be more sensitive than monolinguals to audiovisual incongruence, which was not supported by present findings. This hypothesis emerged from observation of increased attention to the mouth of talking faces (Pons et al., 2015) and increased discrimination of silently articulated foreign languages in unimodal bilinguals compared to monolinguals (Sebastián‐Gallés et al., 2012). Unimodal bilinguals showed no difference between visual scanning patterns for faces articulating audiovisually congruent and incongruent syllables. These results are similar to those of younger monolinguals but differ from those of older monolinguals who showed increased attention to the mouth for audiovisually incongruent compared to congruent syllables. One possible explanation for this unexpected finding might be the increased variability in unimodal bilinguals’ speech experience. Not only are unimodal bilinguals exposed to more than one spoken language, they are also likely to be exposed to more variable models in a given language due to foreign accents. This may be even more important in the present study given that all mothers were bilingual. Therefore, bilinguals are likely to be exposed to a larger variety of audiovisual relationships and could be more tolerant to inconsistencies and imprecision.

The unimodal bilinguals in the present study were a mixed group of infants learning English and one or more other language(s). The target phonemes /b/ and /g/ may be extracted from different phonetic populations for different unimodal bilinguals. A detailed examination of phonetic and phonological differences between monolinguals and unimodal bilinguals is not possible given the large number of alternative languages in the present study and it is unclear how these differences could influence the present results. For this reason, it would be interesting to perform the same task in a more defined group of unimodal bilinguals all experiencing the same combination of languages and phonological systems.

Infants with Deaf mothers experience bimodal bilingualism, and the audiovisual spoken language provided by their primary caregiver is likely to differ in quantity and phonetic properties from that provided by a hearing caregiver. All these factors have undetermined effects on the development of audiovisual speech processing. In the present study, a general increase in looking time to talking faces was observed in bimodal bilinguals compared to monolinguals. Since sign language communication requires visual attention to the signer, Deaf mothers use various strategies to obtain visual attention from their infants (Woll & Kyle, 1989). It was initially hypothesized that this ‘training’ of visual attention in bimodal bilinguals would lead to increased allocation of attention to faces in infancy. However, in the present study, increased attention to faces also tended to be observed in unimodal bilinguals compared to monolinguals, which suggests that the increased complexity of learning two languages (whether they are two spoken languages or a spoken and a sign language) could lead to increased visual attention to talking faces, allowing the integration of additional cues for understanding language.

Moreover, no relationship was observed between bimodal bilinguals’ age and the percentage of time they spent looking at the mouth and eyes of talking faces. This finding contrasts with the attention shift towards the mouth between 4 and 8 months observed in monolinguals and unimodal bilinguals in the literature (Lewkowicz & Hansen‐Tift, 2012; Pons et al., 2015) and in the present study. The shift in attention from eyes to mouth may occur once sufficient audiovisual speech experience is obtained. This stage may be reached by most infants with hearing parents at around 6 months of age, but may occur later in infants with Deaf parents who are exposed to a reduced amount of audiovisual spoken language from their primary caregiver. Studying older bimodal bilingual infants would be necessary to test whether an eyes‐to‐mouth shift is observed at a later stage of development. Moreover, since Lewkowicz and Hansen‐Tift (2012) suggest a link between the production of canonical babbling and selective attention to the mouth of talking faces, it would also be interesting to assess whether a potential delay in this attention shift to the mouth in hearing infants with Deaf mothers is associated with a delay in the production of oral canonical babbling.

The results of the present study also suggest reduced sensitivity to audiovisual congruency in bimodal bilingual infants compared to monolinguals. Indeed, both younger and older bimodal bilinguals showed undifferentiated visual scanning patterns to faces articulating audiovisually congruent and incongruent syllables. This pattern is similar to the one observed in younger monolinguals, yet older monolinguals increased their attention to the mouth in the case of incongruent audiovisual cues of articulation. This suggests an immaturity in the way bimodal bilinguals integrate audiovisual cues of articulation, which could be due to a reduced experience of audiovisual spoken language or an increased variability in speech production from their primary caregiver. Whether this apparent immaturity in audiovisual speech processing in hearing infants with Deaf mothers represents a transient developmental delay in early infancy or the beginning of a different developmental trajectory of speech processing remains to be seen.

Taken together, the results of the present study indicate that early speech and language experience influences the integration of audiovisual speech in infancy. Monolinguals appeared to be more sensitive to audiovisual incongruences in the second half of their first year, while this increase in sensitivity to audiovisual incongruences after 6 months was not observed in unimodal bilinguals and bimodal bilinguals with Deaf parents. Moreover, a shift in selective attention to the mouth of talking faces was progressively observed between 4 and 8 months in monolingual and bilingual infants with hearing parents, while no eyes‐to‐mouth shift with age was observed in infants with Deaf parents who may experience a reduced amount of audiovisual speech from their primary caregiver. We conclude that multi‐modal speech processing in infancy requires sufficient audiovisual speech experience.

## ENDNOTES

1 Deaf with a capital D in this text refers to people who are culturally Deaf, using sign language as their preferred mode of communication and/or identifying themselves as part of the Deaf community.

2 BSL is a natural language with its own grammar, vocabulary and phonology (Sutton‐Spence & Woll, 1999).

3 Silent articulation was dropped from these analyses given the smaller number of trials (*n* = 2) once the other conditions were merged into Congruent (*n* = 4) and Incongruent (*n* = 4).
